# Filtering Touch: An Ethnography of Dirt, Danger, and Industrial Robots

**DOI:** 10.1177/08912416211026724

**Published:** 2021-06-28

**Authors:** Ned Barker, Carey Jewitt

**Affiliations:** 1 UCL Knowledge Lab, London, UK

**Keywords:** dirty and dangerous touch, manual labour, glass manufacturing, waste management, industrial robotics

## Abstract

“Industry 4.0” marks the advent of a new wave of industrial robotics designed to bring increased automation to “extreme” touch practices and enhance productivity. This article presents an ethnography of touch in two industrial settings using fourth generation industrial robots (a Glass Factory and a Waste Management Center) to critically explore the social and sensorial implications of such technologies for workers. We attend to manifestations of dirt and danger as encountered through describing workers’ sensory experiences and identity formation. The contribution of the article is two-fold. The first is analytical through the development of three “filters” to grasp the complexity of the social and sensorial dynamics of touch in situ while tracing dispersed mediating effects of the introduction of novel technologies. The second is empirical, teasing out themes embedded in the sociosensorial dynamics of touch that intersect with gender, ethnicity, and class and relate to the technological mediation of touch.

## Introduction

It is widely reported that collaborative (cobot) and artificially intelligent (AI) robots entering more production processes herald a new industrial revolution (International Federation of Robotics 2019; Pawar, Law and Maple 2016; Sony and Naik 2020; Zamalloa et al. 2017). The rhetorical legitimization of these technological and industrial advancements rests largely on the basis of transforming or eliminating dirty and dangerous work (Royakkers and van Est 2015). There is, however, a lack of research that critically examines such claims and how emerging sociotechnical arrangements are changing the nature of such work.

Grounded in an ethnography of waste management (WM) and glass manufacturing (GM), this article makes a case for the need to better understand how the introduction of novel technologies in industrial settings remediates the sensory tactility and social implications of dirty and dangerous industrial processes. The dynamics of touch that our ethnography attends to are located in, and developed through, three intersecting perspectives that connect the social with the sensory: (1) sociological perspectives on dirt and danger, (2) historical perspectives on labor, and (3) expanded perspectives on touch.

Firstly, we trace the dynamics of dirty and dangerous touch across sensory (e.g., material and experiential) and social (e.g., symbolic) planes (Douglas 1966). Proximity to, and the touching of, dirty and dangerous materials is underpinned by the threat of polluting and harmful physical contact with industrial processes and substances that pose a risk to the health of workers’ bodies. These touches also have implications for laborer’s sensory experiences and social/professional identities. In relation to these we recognize that a wealth of ethnographic literature investigates the sensory character and social implications of “dirty work” (Mccabe and Hamilton 2015; Morales and Lambert 2013; Sanders 2010) and “dangerous work” (Desmond 2006; Johnston and McIvor 2004; Sanne 2008). Vivid ethnographic descriptions of dirty and dangerous work have illustrated the wide range of, often intense or extreme, sensorial inputs that characterize certain jobs and the social dynamics and practices that circulate. These have implicitly shown how dirty and dangerous touch tasks are consigned to particular workforces, gendered, attributed lower status, or are strategically avoided. However, while these studies make clear that *touch matters* in the context of dirty and dangerous work, they seldom bring touch into focus—touch tends to be *filtered* out of ethnographic descriptions.

Secondly, to articulate the benefits of attending to touch in these industrial contexts, we build our approach and argument upon historical perspectives that have analyzed the sociosensorial transformations of labor (Classen 2012; Lindenlauf 2000). Here we recognize that workplaces evolve over time as new technologies are developed. Identities and experiences are remediated as workers duties are reconstructed. While the detailed description of such processes may at first sight seen unimportant, they open up the intricate interconnection between bodies, technologies and environments that is essential to understanding the evolution of the workplace. We locate these historical insights alongside accounts and perspectives drawn from “body studies” (Shilling 2005) and the related subfield of “body work” (Wolkowitz 2006). We join these historical and sociological perspectives together to ground sociosensorial manifestations of dirt and danger within the complex and multidimensional dynamics of touch. Our approach and findings are therefore framed against these literatures through a review of the “tactile histories” of WM and GM. Through this review we expand upon key sociosensorial themes that return from our analysis. For example, these focused industrial histories expose how dirty and dangerous touch intersect with social issues around gender, ethnicity, and class. While articulated with reference to workers’ experiences and identity formation reviewing this literature also reveals that intensities, proximities, and pedagogies are relational to the social and sensory dynamics of dirty and dangerous touch, and further still, that technologies have potential to remediate these relations in *dispersed* ways.

Thirdly, to empirically explore these dispersed technological mediations through ethnography we operationalize an expanded conceptualization of touch that accommodates the sensory character and social implications of touch beyond cutaneous contact and immediate psychophysical affects (Jewitt et al. 2020; Mackley, Jewitt, and Price 2020). This is particularly significant for researching industrial contexts where direct human–robot touch interactions are limited and highly regulated, and where over the course of the ethnography we encountered challenges of “losing touch of touch.” In response we developed three analytical concepts—tactile landscapes, touch trajectories, and touch drivers, to filter and organize data in ways that enabled us to trace and grasp dispersed technological mediations. These three concepts are defined and illustrated through this article.

Against the backdrop of an emerging technological landscape where cobots and AI robots are starting to enter industries, we bring together the three perspectives above through an ethnography of WM and GM. In doing so, this article makes two distinct contributions: (1) it presents an analytical approach to grasp and trace the complex dynamics of touch in specific sociosensorial and technical arrangements; and (2) it offers contemporary empirical insights on how new technologies can remediate dirty and dangerous touch with wider social and sensorial implications. By examining the multidimensional and complex dynamics of dirty and dangerous touch this article illustrates the significant mediating effects of technologies while also identifying continuities that counterweight the revolutionary rhetoric of roboticization.

## Tactile Histories

In this section, we briefly outline tactile histories of WM and GM. Selectively elaborating upon the changing role of dirty and dangerous touch we expose how technologies have come to remediate the sociosensorial dynamics of production as industrial processes have moved from tool-use to a mechanical touch. As will be shown, the fractured transitions from tool-use to mechanized production represents shifts from a direct mediation of touch (i.e., when workers’ tactile experiences and identities are directly shaped by tools) to more dispersed mediating effects (i.e., where workers’ tactile experiences and identities are indirectly shaped by mechanized production). However, despite such revolutionary reorganizations of tactile labor, the classed, racialized, and gendered aspects of the industries, and of touch within them, often appears to be resilient to the advent (and uptake) of new technology. Engaging with these themes demonstrates how dirty and dangerous touch have long been tied to the sensory character and social implications of work in both sectors. Locating this ethnography against these tactile histories continues to frame the sociosensorial perspective of touch upon which this research rests.

### Tool-Use and Touch

Technologies have always mediated touch and danger in glassmaking. Beretta’s (2004) history of glass production from its origins in Graeco-Roman society and throughout history implicitly reveals much about the tactility and sociality of making glass. This included the development of specialist tools to allow a “skilled” glassblower to shape vessels without touching the glass directly. The relationship between tools and touch was an intimate one, honed through the “hands-on” process where tools were an important aspect gaining the requisite *feel* and skills for making glass, as evidenced by Roman glassblowers preference to work with their own tools adapting “their shape to fit the user’s hand (and mouth)” (ibid, 107).

A model of tactile apprenticeship frames glassmaking tool-use. The craft of glassblowing was traditionally practiced and learned in workshops that were both social and productive environments where touch has multiple and central functions. Working with hot and dangerous materials the gendering of dangerous touch was also operating through the regulation of production environments (e.g., women were barred from the glassworks of Murano). Throughout history the skillful touch of craft was translated into occupational identities and traded for social status—that by exclusionary spaces were restricted to certain populations. And while mechanized production has driven a profound reorganization of the tactility and sociality of glass making (as discussed later), the gendering of dangerous touch and its role in identity formation continue in modern industrial settings.

Contemporary ethnographic accounts of glassblowing in hobbyist settings reflect on the pedagogical role of touch. Atkinson (2013, 400) reflected on gaining “a *feel* for the glass and for the tools” where he learnt to touch through tools within a social process. The “instructor’s guiding hand” (ibid, 401) scaffolded the acquisition of tactile capabilities to effectively handle the hot materials. Through apprenticeship the skillful use of tools is a process of embodiment binding to workers’ identity formation (Wenger 1998) in an “assemblage” with tools, fire, and the forming object (O’Connor 2017). These ethnographic accounts also flesh out the sensory intensities (i.e., heat) that frame the experiences of tactile engagements with materials that can burn. Atkinson (2013) and O’Connor (2017) highlight a meaningful amplification of the sensory through the proximities that accompany touching through tools. These contemporary studies raise themes of distance, feel, sensory intensities, and tactile pedagogies as relevant to GM through tool-use. These themes are traced through our ethnography into the modern glassmaking industry.

WM is not typically considered a craft because the industry does not produce goods of consumerist value (although up-cycling is a new craft-practice). Production in this context is to reorder dirty unwanted items for destruction, disposal, or to eventually be remolded into something of value. Lindenlauf’s (2000, 81) sociologically rich analysis of dirt and danger in WM reaching back to Ancient Greece examines how “dirt functioned as a social marker,” in which those who handled it were classified as dirty, wild, and dangerous. Her analysis resonates with Douglas’s (1966) theory of dirt and danger, where symbolic repercussions of touching dirt are socially constructed, and potent. Contemporary ethnographies continue to evidence the social implications of handling dirt for identity formation (Morales and Lambert 2013; Sanders 2010). Waste pickers tend to be immigrants, marginalized, “unskilled,” and lower-class laborers (Yigit 2015). The dirty touch required for waste picking can be unpleasant, dirty, and dangerous (e.g., inhalation of dust—UK Health and Safety Executive 2020), and this can make it an undesirable occupation. As we will elaborate later, while machinery/technology can disrupt the tactility of dirty work (i.e., “sterilizing” or “scaling up” the sensory environment), in WM, it remains common for laborers to sort dirty materials by hand or with basic tools (protective gloves or graspers).

The themes we have drawn out above—sensory intensities, tactile pedagogies, and the othering of dirty-dangerous touch (often across lines of gender, class, and ethnicity) provide the historical backdrop of tool-mediated touch. These histories are both fractured and driven by economic, technological, and social factors.

### A “Mechanical Touch”

The industrial revolution marked the emergence of new configurations of how touch was exploited as a productive resource. In industries, such as GM, a mechanical touch rapidly superseded hand craft and tool-use as the dominant mode of mass production. The pace and extent of transformation were driven by economic forces and the scaling potentials afforded by mechanization. Black (2014) recognized that, for Marx, the industrial revolution gave birth to a kind of monstrous metallic *organism* that sought to bring human bodies into itself. Factories replaced workshops, reorganizing the sociality, tactility, and sensory contexts of manual labor. Among other transformations industrial bodies were “re-engineered” through alterations to rhythms of the working-day (ibid); divisions of labor (Wolkowitz 2006); and exposure to dirt and danger. The social and sensorial conditions of labor were reorganized through developments in technologies, like the glass-pressing machine invented in 1825. For glassmakers touch no longer operated through the tool-mediated feel when crafting objects, rather they now *served* machines ensuring that production lines do not stop and by feeding machines with oil (a manual practice called swabbing). Mechanization across industries distanced laborer’s and their touch from their products in both tangible and abstracted ways (Classen 2012). Moreover, active tactile experiences of tool use gave way to a metallic and unresponsive alien tactility, where the “worker’s touch appeared automatic, almost unfeeling, simply part of the production process. The machine, in fact, seemed to direct the hand, rather than the reverse” (ibid, 180). A new mechanical touch revolutionized industrial tactile regimes and had social implications for the “identities,” “tactile competencies” (skills), and “value” of manual laborers. Black’s (2014) account of embodiment and mechanization details the re-engineering of workers bodies through such technological and industrial developments so as to insert them more seamlessly into a productive relationship with machines—exemplified by the production line.

Mechanization took differing trajectories depending on the nature of the production process, geographic locales, investment, technological developments, and cultural-market trends. A study informant in our ethnography who “lived through this history” of the mechanization of WM shared narratives with the researcher of using shovels to move waste and the hard/dirty labor involved. She also presented the researcher with a copy of “Haulin London’s Rubbish” (O’Connor 2012) a social and technological history of WM. This account of WM depicts the relatively slow uptake of machinery in waste collection and disposal. The partial mechanization of UK picking lines was spurred on by investment into, and growth of, the recycling sector (from the 1960s).

These fractured changes outlined above provide a backdrop to our concern with articulating some of the ways in which the injection of machinery comes to remediate dirty and dangerous touch in industry. The expectations of workers to touch and be exposed to dirt, remain sites of political struggle and industrial regulation made visible through discourse (e.g., trade union disputes), touch is also regulated and contested within the social fabric of factory settings (Wolkowitz 2006). The cultural regulation of bodies, for example, has directed female factory workers to “clean” and “light” forms of manual labor in the UK (*ibid*) and gendered dirty and dangerous touch within industrial settings. In Thiel’s (2007) ethnographic account the historical and modern effects of technology were discussed in terms of how they remediate the dirty and dangerous character of construction, in doing so disrupting manual laborer’s experiences and identities. (We do not have the space to elaborate on these points here; see Stergiou-Kita et al. (2015) for a review of dangerous work; and Thiel (2007) for an intersectional account of dirt and danger on a male-dominated construction site.)

The contemporary industrial moment and the rhetoric around the Industry 4.0 raises questions of how, and to what extent, the emergence of advanced collaborative and intelligent robotics might come to remediate, change, or disrupt dirty and dangerous touch.

### Robotization and Mediated Touch

A new wave of robotics represents a potential to remediate touch by deepening automation and establishing new forms of human–robot collaborations in the industrial workplace. Two major avenues characterize this “new” generation of robotics (Zamalloa et al. 2017). First, advances in AI are allowing robots to operate in more unstructured environments and on more varied tasks. Second, there has been a growth in cobots that work with or alongside humans to increase productivity. These new types of robots are projected to continue to stretch the application of mechanical touch even further into dirty and dangerous industrial territories, they are legitimized on this basis of freeing workers from “unpleasant” and “degrading” touch (Royakkers and van Est, 2015). Strategic government and private innovation funding of advanced robotics signals the momentum behind this shift (Innovate UK 2017). The coronavirus pandemic has only served to heighten attention around issues of dirty and dangerous touch with advancements in AI robotics offered as promising technological industrial solutions to navigate new public health challenges (Kritikos 2020).

Despite the intensification in industrial rhetoric and funding there is a lack of research that critically examines how these emerging sociotechnical arrangements remediate the complex dynamics of dirty and dangerous touch, its sensory characteristics, and social implications. In addressing this gap, this article follows the threads identified throughout this review to investigate (and contextualize) how such technologies might remediate touch in significant ways. Recent developments and uptake of “collaborative” and “intelligent” robotic technologies that assist with dirty and dangerous touches in WM and GM place these industrial contexts at the apex of the themes and tensions that emerged through the critical review above. Consequently, we sought to shed light on these themes through ethnographic research in two “dirty” and “dangerous” UK workplaces—a glass factory and a WM center.

### Methodology

This ethnography builds on a substantial history of work on the sociopolitical consequences of technological development by turning attention to “the body” and its relationship to labor (Cohen 2011; Shilling 2011; Tarr 2011; Wolkowitz 2006). Specifically, the design draws on the concept of “body work” (Shilling 2011) to explore the changing dialectical relationships between body, technology, and labor. Our approach builds *from* the body to focus on touch. We refine this approach by drawing on perspectives from sensory history (Classen 2012; Lindenlauf 2000) and an extended view of touch (Jewitt et al. 2020) to foreground touch within broader social and sensory dynamics. In doing so, we elevate touch through descriptions of workers’ experiences and identity formation. By consistently honing in on the sociosensorial dynamics of dirty and dangerous touch, robotic technologies were not analyzed as “social actors” but considered in relation to their mediating effects (Mackley et al. 2020). Through this approach, the potential of ethnography is harnessed to examine the sociality of automation rather than its politics as expressed through discourse that appears in industrial reviews, White papers, and media. As such, this ethnography responds to Bissell and Del Casino’s (2017) call for research to closely examine how new robotic technologies change the nature of work.

### A Multisited Sensory Ethnography

The article reports on a sensory ethnography on the social implications of robotic touch in industry conducted across two sites (overview in Baker et al., 2020). Robotic touch as it appears in “the real world” consists of a set of highly differentiated events that are “spatially dispersed” and cannot be researched as a homogenous phenomenon. This lends itself to an approach, which involves ethnographers undertaking research in different physical locations as part of a single study (Falzon 2016). This article presents data that coalesces around the substantive themes of dirt and danger. The social and sensory dynamics of dirty and dangerous touch are intensified in WM and GM—supported by the tactile histories presented earlier.

**Figure 1.**
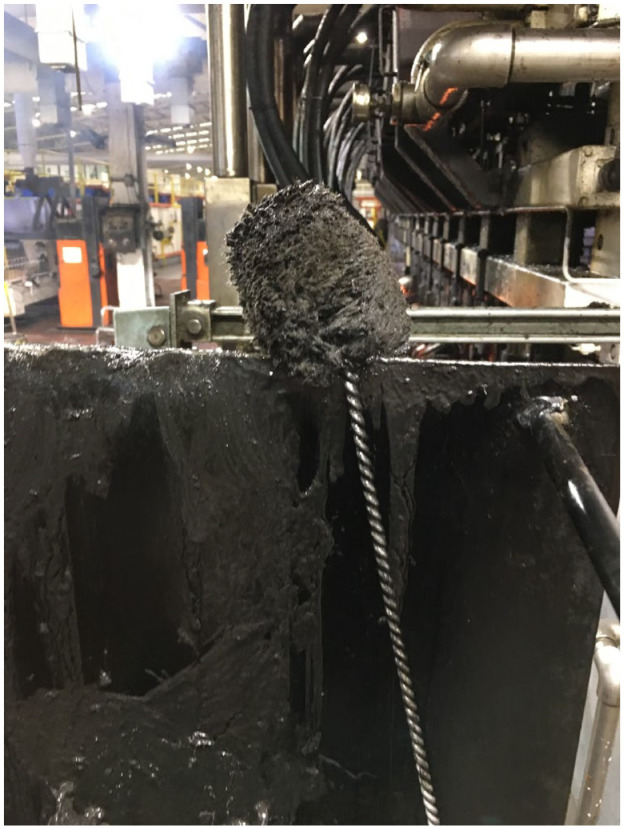
A swabbing brush/stick used to apply oil (dobe) to the molds in the machinery that can be seen in the background of this photo.

The GM site had recently installed cobots to work alongside “hot end” operators (herein operators) in “swabbing” the machines that mold molten glass into bottles. Swabbing was once an entirely manual job that involves brushing oil onto the molds. There are health and safety regulations/procedures that regulate swabbing practices, but due to the heat and power of the machines it is still considered a potentially dangerous touch task. Accidents can occur, for example, one operator explained that swabbing sticks (Figure 1) can get stuck and “go up in flames straight away”. Socabelec (2020) stated that their cobot was “born” in 2013, to “change and improve the health and safety of workers operating the ‘swabbing’ section at their factories, a difficult and hazardous part of the workspace” (n.p). At the GM site the cobot will swab one side of the molds while the operators do the other, *automating* up to 50% of this “potentially dangerous,” hot, and smoky touch task.

The WM site had recently employed an AI sorting robot, Max-AI AQC (herein Max). This robot is advertised as delivering an “overarching brain capable of expanding labour free automation and *optimisation*” (BHS 2020, n.p). Max is able to “learn” how to accurately optically identify, and pick, disordered and dirty waste materials. This AI sorting robot was added to a new section of the picking line—providing yet another technological addition to a process that already combines various manual and mechanized methods to sort waste materials.

The field researcher spent one week in each site, shadowing shift patterns of manual laborers. Participation (wherever possible) was a cornerstone of our approach: (1) to avoid the potential sterilizing impact of methodological distancing (e.g., relying too heavily on observation, conversation, and interviews) because we note that many contemporary ethnographies of “dirty work” keep the researcher at arm’s length from dirt (Mccabe and Hamilton 2015; Morales and Lambert 2013; Sanders 2010); and (2) as touching and being touched is essential in “bringing the ethnographer closer to sensory and semiotic action” (Barker, Jewitt, and Price 2020, 129). Participation took the form of attending training; laboring alongside workers and robots; taking breaks and having conversations with workers; talking about touch (and other sociosensorial matters) with laborers during activity.

**Figure 2.**
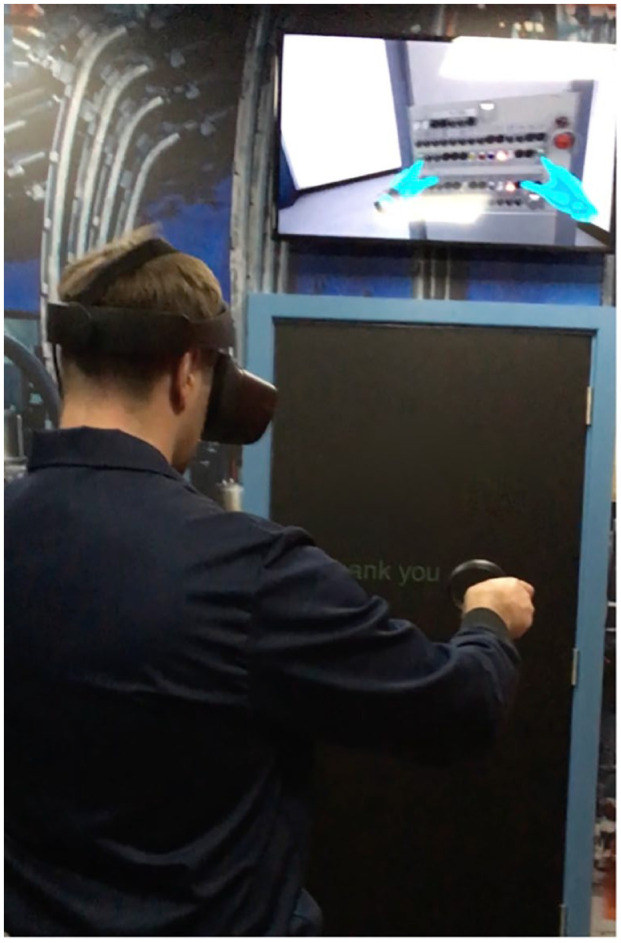
Field researcher learning how to swab using touch controllers in virtual reality.

A participatory approach was pursued through tactile apprenticeship, inspired by the ethnographic tradition of developing knowledge through apprenticeship, and refining Pink’s (2012) “sensory apprentice” to touch. As part of negotiating access permission was sought for the field researcher to labor alongside workers and robots. The aim was to enter the field and collect data from the viewpoint of a tactile apprentice, learning how to touch with others and machines. This was challenging at times, complicated through specificities of dirty and dangerous touch in each site. In the WM site, the researchers’ motives to touch dirty objects were seen through suspicion and bemusement, positioning the researcher as an outsider, and presenting a cautious barrier between researcher and pickers as they worked together. Participating in dangerous touch was strictly regulated at the GM site. The ethnographer was under instructions that “you cannot touch anything to do with the machinery” after arrival and induction. Operators demonstrated and supervised tactile experiences, including manually checking the quality of a bottle on cooled glass; and “swabbing” in the virtual reality training simulator (Figure 2).

The noise levels in both sites meant that verbal communication was at times difficult and posed problems for video recording. This also afforded research opportunities because much communication occurred through tactile cues. Added to this was the challenge of balancing and foregrounding touch while appropriately situating it within the sociosensorial context. Sensory fieldnotes based on participant observation (with an emphasis on participation) and tactile apprenticeship were collected. These were supplemented by data collected through videographic and photographic records of guided walk-throughs following the production process and (audio) recorded semistructured interviews (eight interviews lasting between 30 minutes to 1.5 hours).

### Analytical Framing

First contact with touch in the field raised the challenge (methodological and analytical) of how to track the presence and absence of touch and the mediatory effects of technology in industrial contexts where direct contact between human and robot was often limited and regulated. The replacement of workers’ dirty and dangerous touch was the immediately visible implication of both technologies. Through a broad understanding of touch and its mediation as dispersed rather than direct, the ethnography was able to respond to and trace the sociosensorial dynamics of touch. In response to these challenges we developed three analytical concepts. We drew on tactile metaphors, applying “filters” (tactile landscapes, touch trajectories and touch drivers—introduced below) to handle the data gathered through our first contact with the sites. We refined and operationalized these filters further to assist and structure data collection and analysis.

#### Tactile landscapes

Touch is more than points of physical contact. Touches come and go in the unfolding sensory engagement with workplaces—dull or intense, meaningful, or inconsequential. These unfolding tactile experiences provide a landscape that embodied workers (and the ethnographer) move through as part of the production process. Methodologically we mapped these tactile landscapes by sketching its outlines and detailing its contours. The ethnographer moved through the full production process and range of environments (e.g., on guided tours, following the production line, going on collection runs with dust-cart drivers), touching and being touched where appropriate. The ethnographer was drawn to particular locations to detail touch where it was at its most dirty and dangerous and to areas near the robotic technologies. From these more fixed positions, data collection was led through sensory participation and tactile apprenticeship to contour the landscape and understand touch in relation to workers’ experiences.

#### Touch trajectories

Touch is not static. This was the case when tracing historical relationships between touch, dirt, and danger. Workers come to learn through touch and how to touch. The social and sensory environments contain tactile pedagogies. Through this filter, we sought insights on how dirty and dangerous touches were experienced and made sense of by workers over time. Semistructured interviews were designed to enable laborers to articulate how their (social and physical) relations of touch had developed through dirty or dangerous work. Field observations were incorporated into these interviews to open up discussion around areas of thematic and tactile interest. Furthermore, we used the relatively novel appearance of new robotic technologies as tangible discussion points to consider how these trajectories change or persist. We examined how touch was drawn on in the formation of social identities and marked trajectories toward professional status.

#### Touch drivers

This filter seeks to capture and critically explore what drives the formations/dynamics of the tactile landscapes and what shapes touch trajectories within and beyond industrial contexts. Two types of ethnographic encounters were important in assisting these inquiries: (1) communicating with managers during a joint observation of a touch task asking “why it is done like that” and (2) documenting and analyzing culturally emotive exchanges where articulations of dirty and dangerous touch were present and contested. Emotive or charged exchanges were revealing as they contain power relations where driving forces can be analyzed, in both sites these forces were characterized as social, political, economic, cultural, and technological.

Collectively these three filters recognize the social and sensory implications of touch as they reverberate across disparate configurations of time and space. They were developed in response to the complexities of the fieldwork, and to navigate the challenge of “losing *touch* of touch” and its mediation.

## Filtered Touch

The fieldwork is presented through the tactile landscapes and touch trajectories filters described above to examine the mediating role of novel robotic technologies (the WM AI sorting-robot, the GM swabbing cobot). The sociosensorial dynamics of touch are largely expressed through illustrations of workers’ experiences and identity formations. Key social, cultural, and economic drivers are threaded through the discussion.

We focus primarily on the WM site in tactile landscapes to describe how touch is *experienced* across the production process. In touch trajectories, the GM site is foregrounded. In both sections key themes are fleshed out with selected illustrative examples from the other site to develop an awareness of the complexities of touch. The technological mediation of dirty and dangerous touch as they are captured and expressed through both filters concludes each section. In the next section, we discuss tactile landscapes through themes of intensities-sensitivities and positions-divisions, before tracing technological mediations.

### Tactile Landscapes: Intensities-Sensitivities

To render fine-grained accounts of the contours of the tactile landscape, we filter experiences of touch within the pre-pick cabin (WM site) and the “hot-end” (GM site), which workers described as the “dirtiest” and most potentially “dangerous” areas of the production processes and where the most intensely experienced forms of touch were encountered.

*Fieldnote WM site, encountering intensities*: There was an experiential baseline to the tactility of picking and sorting the waste in the pre-pick cabin—a palate of familiar smells and touches. Yet, there were moments when touching the discarded materials felt riskier and/or more unpleasant. The smell and sight of organic materials sometimes arrived in putrid concentrations, one informant used “shitty nappies or dead pigeons” as examples of particularly unpleasant encounters. These illustrative materialties represent an intensity of “dirt as decay” where direct touch might be avoided by the pickers or performed with assistance of other available materials as makeshift tools. These subtle innovations in picking practices using techniques and “tools” aim to physically distance decay from the hands and nose. While actively avoided, bodily boundaries could sometimes be unexpectantly exposed. Today I grabbed a concealed item, instantly feeling an unidentified cold liquid seep through the protective glove. This experience punctuated my awareness. I recoiled, and inspected the extent to which I was exposed to dirt. During the same shift, a number of syringes (unused and needle-less) passed by—my picking movements became more cautious as I scanned for potentially hazardous waste. My embodied response as a tactile apprentice was that picking momentarily felt more dangerous, or risky, as my bodily boundaries were made vulnerable through imagining touching dangers hidden in the disordered materials.

This fieldnote illustrates how tactile experiences of picking fluctuated in response to bodily encounters with a materiality and sensorium in motion. Two main considerations framed the intensity of dirty touch, these were concentrations and closeness. On a material axis, concentrations of decay (as sensed through aromas, touch, and sight) fluctuated, continually elevating or depressing experiences of performing dirty work. On another axis, perceptions of how close the dirt feels to one’s body informed the sense of exposure to waste. Together these fluctuating dimensions brought the intensities of dirty touch to a sense of risk or danger. In the GM site the intensity of the sensorium when in touching distance to sources of power and heat (i.e., the machines or furnace) added to the sense of danger of coming into contact with the machines and/or materials. Dirt was also connected to danger in this industrial context, with workers “housekeeping duties” requiring them to continually clean away oil, broken glass, and tools to ensure a safe workspace. These examples show material dynamics of “dirt as disorder” and “dirty as decay,” that are connected to danger, and leave symbolic marks on those who come into contact with them (Douglas 1966).

In both contexts, there was evidence that encountering intensities of tactile experience (that manifest as both dirty and dangerous) could be somewhat tempered by a process of desensitization. Over time the field researcher recorded in their reflexive diary that they were becoming increasingly desensitized to the aromas and tactile feedback from touching dirt in the WM site. In the hot end two operators discussed how the heat and prospect of coming into contact with the machines is initially “daunting” but that this sense of fear erodes over time to a point where “now I’ve been working with them [the machines] for years I just look at it” (established operator).

The intensity of workers experiences of dirty and dangerous touch was connected to three interrelated factors: the concentrations of sensory inputs (e.g., heat and aroma); the bodily closeness to dirty-dangerous materials; and the embodied sensitivities to these material dimensions.

### Tactile Landscapes: Positions-Divisions

This section considers the driving forces that situate particular types of workers (e.g., human–robot, male–female, skilled–unskilled) across the dirty and dangerous touch terrain. In all corners of both tactile landscapes there are stark distinctions to be made depending on where workers are situated and what the manual touches they are employed to do. In WM, the composition of materials that were handled varied between the three sections of the line. After waste and recyclables have been removed the “cleaner” and “lighter” objects remain further down the line. Pickers toward the end of the line are expected to sort at a faster rate. Working on each section elicited different sensory-tactile experiences, expectations, and surveillance methods (e.g., “pick-rate” [number of picks per minute] being measured occasionally through CCTV footage). Divisions of labor that were a result of placing workers in “suitable/productive” sensory and tactile environments. According to one informant at the GM site there are at least “two very different specialisms” and sensory environments in the glass factory: the hot-end where bottles are formed and the facility where they are filled and packaged. The former is “dirty, hot, and oily” and requires a specific set of technical and manual skills that were described as oppositional to the hyper-sterile and temperature regulated environment of the later.

Economically driven calculations rationalized the increased automation to the line and the positioning of employees, machines, and robots across these varied landscapes. This economic agenda sought to maximize each of their capacities for touch as a productive resource, often assessed in quantified or monetary terms. At the WM site, the cost of a pickers’ touch (to remove something from the line) was estimated to be “about 1p,” and the sorting robot was considered valuable if its touch could cost less. The shape, contours, and divisions of the tactile landscape, therefore, were backdrop to economic evaluations around the dirty and dangerous touch of humans *set against* productive potentials of robots. Such calculations informed decisions around how these technologies are integrated or appended to the line. Furthermore, observable patterns of division with respect to how laborers were positioned across both tactile landscapes were articulated through gender and cultural norms around touch.

Sociocultural positioning orientated around perceived distinctions of touch aptitudes and capacities based on ethnicity and social class, as well as comparison between the relative productive value of human and robotic touch. Uncovering these influences exposed power relations around who is (or is not) protected from dirty and dangerous touch. For example, the gendering of particular forms of touch intersected with economic considerations/rationales to position workers across these tactile landscapes. The workforce in the WM pre-pick cabin and workers in the hot-end were overwhelmingly, if not exclusively, male. The former driven by cultural assumptions about capacities to touch, and sensitivities to touch. Male staff were assigned to the cabin for two stated reasons: “women tend to be better at multitasking” and suited to sorting multiple materials on the “mainline”; while men “don’t seem to mind picking through the waste at its dirtiest and are less bothered by the cabin smells.” The “lads,” a term used to refer to hot-end operators, gendered the space and touch within it. This gendering remained implicit, although the contours of the tactile landscape in the hot end gave plenty of opportunities to practice, and in doing so reinforce, such social connections between dangerous touch and masculine identity (Stergiou-Kita et al., 2015).

### Technological Mediation of Tactile Landscapes

The extent to which the technology (i.e., the swabbing cobot and AI sorting robot) mediated the tactile landscape of each site varied, as did the tangible material effects for workers experiences of dirty and dangerous touch. A key factor was the positioning of workers in relation to the intensities of the sensory environment and the repositioning that sometimes occurred because of the introduction of Max or the swabbing cobot. A significant distinction framing these tangible mediating effects was robot-as-appendage (WM) versus robot-as-integrated (GM).

Robots, such as Max at the WM site, that are *appended* to a line barely fed back onto it in material ways. This resulted in Max being put to work in relative isolation meaning it did not significantly alter what or how workers touched. Pickers material encounters with dirt and danger or the intensities of their tactile experiences therefore remained stable. Positioning Max as appendage was partly driven by health and safety regulations (to avoid dangerous collisions), however, the economics of touch as a productive resource was the main driver. The company experimented when they first incorporated the sorting robot and evidenced that was uneconomical to place pickers after it. Furthermore, they reasoned that by isolating the robot from the mainline robust cost-benefit evaluations are possible where they can monitor its “pay back” period and compare to manual pickers.

While the direct and tangible mediating effects of the tactile landscape are currently minimal, the appendage of Max disrupted the ways in which touch worked and raised questions around the potential for, and social implication of, replacing dirty touch. It brought comparisons between the capacities of human and robotic touch into awareness, and stimulated internal discourse with particular attention to the scaling of dirty touch. Time spent picking was a point of comparison “humans need breaks, whereas Max doesn’t. He could potentially touch stuff for more than eight hours a day” (Managing Director). The company showed the field researcher future plant designs that enable robots to pick unassisted “24/7” to process more materials while extracting more recyclables. The appendage of Max stimulated possibilities and imaginaries around creating distance between bodies and waste, “the intention is that humans move away from being near and touching these dirty materials and are employed to do quality control” (Managing Director). This redrawing of workers positionalities imagines a significantly changed tactile experience and profiles of the work, and stimulates pickers potential anxieties at change, akin to neo-Luddism (Nam 2019), which circulated in organizational conversations.

At the GM site humans and cobots worked alongside each other sharing the same workspace. Usually the cobot would automatically swab one side of the mold (it is currently limited to one side), and the operator would swab the other. When schedules aligned, both would perform their respective task in unison. The sharing of tasks and space represents an *integrated* model. Here the tangible mediation of the tactile landscape can be felt and traced, as it means the operators spends less time manually swabbing, and time close to the machines. The cobot therefore reduces workers exposure to the intensity of the heat and smoke. This had implications for workers tactile experiences working in the hot-end, and the fast-paced nature of attending to the machines. The integration of the cobot subtly rebalanced the tactile duties performed, and competencies required, to work in the hot-end. This tangible remediation of the tactile landscape was tangled to identity formation through the themes of time and tactile competencies.

We now turn to describe the specificities of the touch trajectories formed through an engagement with tactile landscapes sketched above, with attention to the importance of danger and heat to the trajectories of operators at the GM site, and dirt in the case of the workers at the WM site.

### Touch Trajectories: Learning through Dirty and Dangerous Touch

Established hot-end operators raised the process of “learning through touch” and the importance of touch in gaining a *feel* for the machines, materials (glass in its various states), and tools: “If I’m training someone and I go, ‘you press this, you press that. . . You touch this, you move this.’ If you don’t really do it yourself you’re not going to get the feel of the machine” (established operator, A).

Learning through touch and gaining a feel the production process was discussed as necessary across all aspects of their tactile duties, the use of tools and the (glove protected) hand directly during manual quality inspections, “when we check the shape of the bottles a lot of it comes down to feel” (established operator, B) and “we have metal gauges . . . [but] I haven’t touched the metal gauges because I know I don’t need to, I’ve felt it. I’ve gone, yep I’m happy with that.” (line specialist). The perceived value of human touch was referenced against the ability to handle uncertainties within the manufacturing process, or to gain a feel for the “dark-art” of glassmaking. Gaining a feel was on a tangible level a process of learning through touching, and on a social plane it was tied to the formation of professional identities.

Gaining this feel, however, was framed through danger and heat which regulated workers bodies and physical interactions with the machines and the intensities of the tactile landscape (e.g., heat, pace, power, noise, and forces): “You have to be a bit cautious because you are not going to just start touching random parts of the machine or buttons. But yeah, you learn through touching” (established operator, A). To train tactile competencies new starters begin by practicing techniques (swabbing, changing parts) and “learn[ing] exactly what to do with the hands” in virtual training rooms. They practice on physical replicas of the machinery in conjunction with virtual simulators, which approximate and simplified touch through haptic controllers (Figure 2). As the comment below from a new starter reflects that this sterilized training provides useful knowledge but offers limited exposure to the intensities of the tactile landscape against which experiences of dangerous touch emerge:

[attending to the machines is] dangerous init. You don’t know what you are looking for, so to see it on the VR it’s kind of useful. You can’t get used to the heat, but it gives you a good idea (new starter, 4 months into their training).

Once starters graduate to the “shop floor” (wearing an orange cap to signal their status) their tactile apprenticeship continues under the “guiding-hand” of a supervisor. Operators comment on other markers that make the starters stand out. These are based on how they *handle* the intensities of the tactile landscape including being prone to drop tools because they felt too hot; start to panic if something gets stuck; responding to time-pressured events by freezing or rushing; fixating on the task at hand and ignoring periphery senses that signal “something is up” (composition of points made three established operators). Gaining a feel for the production process, through touch, in concert with being able to *handle* the intensities of the tactile landscape in defined ways were central resources in forming operators’ social and professional identities.

No mandatory training was required before handling dirty materials at the WM site. Pickers begin to learn through touch after completing a site-specific health and safety induction and putting on personal protective equipment (PPE). However, picking effectively (fast and accurate) is a coordinated, reactive, and skillful activity. The field researcher learned picking techniques that relied on an attuned tactile feel through touching waste, copying coworkers, and from generous guidance offered through their gestures. We extend the notion of “feel” usually reserved for high-value (e.g., craft) to “low-status” tasks in these marginalized forms of production. The tactile and sensory properties of the waste informed decisions and movements of the tactile apprentice/researcher. For example, physical contact with objects provided a basis to judge the quickest way to split a bag and spread the contents. Tactile explorations mingled with aromas of organic matter to sense the contents of an opaque bag, influencing decisions whether to rip it open, or not. Learning through touch was an important process of attunement in this industrial context where optics can deceive both human and robotic pickers. Many plastics look like paper and pickers constantly employ touch capabilities to gather feedback on materials, using tactile-audial criterium such as “if it rustles let it go, if it doesn’t pick it” to inform decisions.

We point to some of the complexities around becoming “skillful” and “experienced” touchers that recast considerations around the social and sensorial dynamics of dirty and dangerous touch.

### Touch Trajectories: Embodied Relations to Dirty and Dangerous Touch

Learning through touch led to alterations in workers’ embodied relations touching dangerous and dirty materials. The examples below expose intimate dynamics between the sociality and sensory tactility of the professions. For work*men* in the hot-end, social value was attached to regulating and controlling instinctive reactions to touching hot and dangerous materials, tools, or machines. For work*men* on the picking-line the transaction between time spent picking through dirt and wage was elevated.

Tolerating heat was not only a necessity to being a “productive” worker it was central to the sociality of working in the hot-end, and a key aspect of identity formation within this male dominated space. One operator recounted that “I got made fun of when I just started, being called *fairy liquid hands*. . . I couldn’t hold any tools that long without burning.” A culture of been able to “handle the heat” framed how another operator attempted to regulate their reactions to holding hot objects, against instinct and training advice, “when you start you are told to drop something if it is hot—but you wouldn’t want to because you don’t want to *lose face*.” This operator continued to articulate that “when you first start you don’t know your limits around how long you can hold tools for, but over time you know.” While this touch trajectory is, in part, based on gaining an awareness of durations and increasing tolerances when proximal to, or in contact with, intensely hot objects, it also relies on a process of tactile desensitization through exposure and repetition (see tactile landscapes). The social dynamics of the working environment reinforced these trajectories, with implications for how hot objects were handled as well has how workers *handled* the heat. These changing embodied relations to touch were also prerequisites for gaining a feel for the machines and tools.

It was not the ability to handle the dirt or aroma that held social value at the WM site, rather tolerating the dirtiness of the waste, becoming desensitized to it, was merely viewed as a requirement for continued employment. In contrast to the sociality of dangerous touch in the hot end, there were few social gains from handling dirty materials, as dirty touch rarely contains social prestige (Douglas 1966). This was recognized by office staff who suggested that the unpleasant and repetitive nature of the work made recruitment and retention an industry-wide challenge in the UK. Despite claims that pickers are “relatively well paid,” domestic employees (UK citizens) would “only last two weeks max” (office staff). These cultural assumptions of who can “handle” touching dirt or tolerate it for the financial return on offer, expose ethnicity, and immigration status as factors in assuming who potential employees might be and what their entry dispositions toward dirty touch are. The absence of cultural and social capital being generated through dirty touch exposes the heightened importance of the transaction between wage and the manual labor of dirty work. The transactional nature of dirty touch and the internal politics that surround this serves to deepen distinctions and divisions within the industrial settings. This finding is in accord with Iverson’s (2020) ethnography of “canning” in a recycling center because office staff frequently moralized the dirty work through environmentalism (through which Max was positioned positively), and yet pickers did not appear to engage with, or draw value from, such virtuous connections to reframe their relation to dirty touch.

### Technological Mediation of Touch Trajectories

The appendage of Max mediated touch trajectories as situated in the sociality of dirty work by stimulating comparisons between the touch and learning capacities of human pickers versus robots. Through different processes both Max and pickers “learn” through touch and their “senses.” When brought into the same space and production process, the workforce perceived the productive possibilities and limitations of both forms of touch through a comparative frame. Pickers retained the advantage through their ability to learn to select materials more intuitively, perform complex picking motions, and *feel* the waste to increase pick rate and accuracy. In contrast, Max’s lack of tactile sensors and reliance on sight to identify and pick materials were limited (e.g., Max struggles to learn to distinguish between cardboard and wood) and perceived as a disadvantage. Imaginaries of the future organization of the picking line that were aired in public workspaces would often draw upon nationality and immigration status, exposing the social dynamics around the othering of dirty touch uncovering cultural forces that shape perceptions of who it is for, and who it is not. These public exchanges reveal changing relations of labor value and power (as stimulated by the arrival of a new technology and its potential future uptake). The othering of dirty touch also speaks of the sociosensorial continuities (e.g., social and power inequalities) within WM that remain stable in spite of the possibilities for new technologies reorganize the industry.

The integration of the cobot at the GM site had more tangible mediating effects on the embodied processes of learning through, and learning to, touch. The status of the nature of collaboration between worker and cobot was ambiguous. One operator’s response to the question “do you work with or alongside the cobot?” reflects this ambiguity, “[it’s] a bit of both, like you do your own work but you do have to work together.” In practical terms, as observed, there would be periods where operators would “let the robot run itself really” and occasions where they would align/modify their schedules so that they can swab both sides of the mold in unison. The collaboration that was observed and discussed was one led by workers.

More generally, the swabbing cobot was discussed in terms of altering the operators working days with the technology allowing them “more time to step back and see the bigger picture” (established operator, B). In addition to having temporal effects on the learning process, this duration at distance from the heat of the machines has implications for operators experiences and their sense of dangerous touch. Two operators talked about their health and well-being being improved as a result of reduced exposure to the heat and breathing in the smoke (which blows into their faces while swabbing). Consequently, the cobot was seen in a positive light among operators, “[I welcome] anything that gets your body away from the heat and machinery” (established operator, A). This trumped the initial concerns operators had when they were required to learn how to swab at twice the speed. Coming to “handle the heat” as a social and tactile learning process was therefore remediated through the cobot in terms of operators relations to time/pace, heat, and danger. One social implication of the cobots being integrated into a shared workspace and sharing the touch task of swabbing with the operators were articulated through a process of learning to trust the robot. Articulated through workers’ experiences we note that the type of *trust* that formed through interacting, touching, and swabbing together with the cobot was neither bidirectional nor mutual. Rather we examine the users (operators) trust in the cobot and suggest that this was initially centered on the perceived performance and proximity of the cobot—and echoing Maurtua et al.’s (2017) findings our participants described how degrees of trust changed over time and familiarity.

Operators spoke of their *initial* hesitancy at moving into the path of the cobot in case they were harmed by a collision. According to one operator it did not take long before he did not “think about it [unintended dangerous physical contact with the machine] anymore.” This trust was established quickly and was considered to be a decisive factor that set the robots apart from the machinery, “machines would just keep going. . . so if I stick my hands in the blanks [molds] it would just keep closing on my hand, it will not stop. Whereas robots are much more advanced and aware of you, less likely to hurt you.” Beyond this initial phase humans developing trust that robots will not hurt them, the term trust was also connected to perceived performances and how the cobot could impact upon how the worker might be evaluated by management. The formation of this type of trust was complex and took time to build. For example, one operator identified trust as an issue with the robot because “at the end of the day you are responsible. If the robot is swabbing it incorrectly and later down the line defects are being picked up, then you are to blame.” Learning to trust cobots intersects with decisions of whether or not to manually swab therefore became part of a new touch trajectory. A line specialist (C) stated that after a while he realized that the cobot “makes me look good because all my targets are higher. You know for that job I used to pack in around 84/85[% rate of acceptable bottles], and now if I don’t have a 90[%] I’m not happy.” He continued to describe how from this he came to “love” the integration of the cobot, naming “her” Deborah. Naming the cobot (or indeed assigning Max “parents” in the WM site) did not attribute human qualities to the technologies in any meaningful way in these industrial contexts. They were not perceived as elevated to an equal coworker, nor protected against dirt and danger through legislation or other means. Instead, the personalization and naming of robotic technologies demonstrates how they remediated the social (as well as sensorial) ecology (Mackley et al. 2020) of that industrial setting. Navigating this new territory has potential consequences for how, and what, operators learn through touch.

## Conclusion

The contribution of this article is two-fold.

First, it contributes an analytical approach to tracing technological mediation developed in response to the complexities of exploring touch through fieldwork, notably the challenge of avoiding “losing touch of touch.” As part of this approach, we introduce and demonstrate the use of three analytical concepts—tactile landscapes, touch trajectories, and touch drivers, that can support an extended perspective on touch. Collectively these filters offer an analytical route to recognize the social and sensory implications of touch as they reverberate across dispersed configurations of time and space. This approach has analytical power for future ethnographic work on touch and qualitative research on touch more generally, beyond the specifics of the study of dirty and dangerous touch in the context of industrial labor. Future irritations and applications of these filters in different empirical fields will require the concepts to be newly refined (e.g., in terms of spatiotemporal (re)configurations of human–machine interaction). While acknowledging the methodological and empirical challenges of this filtration process (e.g., balancing and foreground touch while locating it within sociosensorial context), we contend that refining a focus *from* the body to a concern with how touch works provides an avenue to grapple with the complexities of technological mediation. As demonstrated in this article filtering touch does not separate tactility from other senses nor from the industrious and social body (Shilling 2005) rather it redirects attention to how touch works, adding another dimension to the felt realities of automation (Bissell 2020). Our analytical process made accessible the technological mediation of touch in settings geared toward hyperproduction at scale, giving voice to important power inequalities that are fused to dirty and dangerous touch.

Second, this article contributes empirical sociological insights on the tactility of industrial labor. By examining the nitty-gritty sociosensorial dynamics of touch in industry, we have shown *continuities* that stand in contrast to the revolutionary rhetoric of robotization (Pawar et al. 2016). The discussion detailed the stability of culturally and historically situated frames that ensured the othering of dirty-dangerous touch persisted in spite of transforming production processes. In WM the dismissal of tactile competencies (or skills) of the pickers exposes the dirty mark left on the social status of the pickers (connected to gendered and migration discourses) that has been identified since Ancient Greek societies (Lindenlauf 2000). In the hot-end the social value of learning to handle the heat operated to reinforce social connections between dangerous touch and masculine identity (Johnston and McIvor 2004). The article also identifies some significant disruptive technological *remediations* regarding the social and sensory dynamics of dirty and dangerous touch. In both sites, differently so, the presence of AI and collaborative robots became part of the ecology that encompassed workers professional and social identity formation. This mediating effect is not dissimilar to those of the relations between touch and tools or machines in industrial settings. However, by tracing the mediating effects of these robots newly formed issues around trust (GM) and anxieties (WM) were exposed and related to sociosensorial manifestations of dirty and dangerous touch.

To conclude, based on the assumption that industry 4.0 will continue to stretch further into dirty and dangerous processes we argue that contemporary ethnographies, such as the work presented in this article, make a significant contribution by taking strides toward understanding and addressing the sociosensorial implications of how touch is changing beyond its economic and productive outputs.
